# Development of a Smart Signalization for Emergency Vehicles

**DOI:** 10.3390/s23104703

**Published:** 2023-05-12

**Authors:** Muhammad Hameed Siddiqi, Madallah Alruwaili, İlhan Tarimer, Buse Cennet Karadağ, Yousef Alhwaiti, Faheem Khan

**Affiliations:** 1 College of Computer and Information Sciences, Jouf University, Sakaka 73211, Saudi Arabiaysalhwaiti@ju.edu.sa (Y.A.); 2 Department of Information Systems Engineering, Muğla Sıtkı Koçman University, Muğla 48000, Turkey; 3 Department of Computer Engineering, Gachon University, Seongnam-si 13120, Gyeonggi-do, Republic of Korea

**Keywords:** emergency vehicles, signaling, smart traffic lights, smart roads

## Abstract

As the population increases, the number of motorized vehicles on the roads also increases. As the number of vehicles increases, traffic congestion occurs. Traffic lights are used at road junctions, intersections, pedestrian crossings, and other places where traffic needs to be controlled to avoid traffic chaos. Due to traffic lights installed in the city, queues of vehicles are formed on the streets for most of the day, and many problems arise because of this. One of the most important problems is that emergency vehicles, such as ambulances, fire engines, police cars, etc., cannot arrive on time despite traffic priorities. Emergency vehicles such as hospitals and police departments need to reach the scene in a very short time. Time loss is a problem that needs to be addressed, especially for emergency vehicles traveling in traffic. In this study, ambulances, fire brigades, police, etc., respond to emergencies. A solution and a related application have been developed so privileged vehicles can reach their target destination as soon as possible. In this study, a route is determined between the current location of an emergency vehicle and its target location in an emergency. Communication between traffic lights is provided with a mobile application developed specifically for the vehicle driver. In this process, the person controlling the lights can turn on the traffic lights during the passage of vehicles. After the vehicles with priority to pass passed, traffic signaling was normalized via the mobile application. This process was repeated until the vehicle reached its destination.

## 1. Introduction

Traffic is one of the most widespread problems in the world. Traffic includes pedestrians, vehicles, riding and farm animals, trains, and other vehicles that use the roads for travel and transportation. Population growth in urban areas has undoubtedly led to an increase in the number of vehicles on the roads and, hence, an increase in traffic problems. However, traffic management techniques are being used to avoid these problems through the Internet of Things (IoT) [1,2] and ad hoc vehicle networks [3,4]. The most common and effective traffic management techniques include speed bumps, road closures, turn restrictions, traffic signs, raised pavements, signaling systems, and electronic monitoring systems.

Most problems are related to highways, the most widely used mode of transport in the world [5]. Traffic accidents are one of the main problems on motorways. Most of these accidents occur on motorways in residential areas. The causes of these accidents in residential areas include traffic congestion (heavy traffic), inadequate technical infrastructure, and inadequate structures such as underpasses and flyovers. The main causes of traffic congestion, which ranks first among the most important causes, include inadequate public transport, unsuitable parking, road works, accidents, excessive traffic on the same route, and too many pedestrians. Vehicles, apart from the rude behavior of drivers, are also a major cause of traffic congestion. Due to this and similar behavior, emergency vehicles such as ambulances and fire brigades, where safety and timing are important, face major problems.

Another important reason is the lack of technological infrastructure. The most important elements used to manage traffic in settlements are traffic light signaling systems [6]. Traffic lights are signal devices placed at road intersections, pedestrian crossings, and other locations to indicate that it is safe to drive, ride or walk. Signalization systems are divided into two basic groups according to their working principles. These are isolated systems and coordinated systems [7,8,9,10].

With the increase in population worldwide, the number of vehicles in traffic is also increasing. This situation brings along many problems. One of the most important methods to reduce these problems is smart signaling systems. Many researchers have tried to make traffic smoother by developing various methods in this regard. Intelligent signaling systems prevent traffic density, accidents, etc., as well as minimize time losses. Time loss is an important problem that needs to be addressed, especially for emergency vehicles traveling in traffic. Every second counts for emergency vehicles. Traffic jams on signalized roads greatly hinder the speed of emergency vehicles. Although these vehicles have the advantage of running red lights, they are not safe and heavy traffic prevents them from doing so.

The purpose of all signaling systems is to reduce the likelihood of accidents and reduce delays by ensuring that traffic flows pass uninterruptedly and without following each other and by providing control at uncontrolled intersections. Even when signalization systems are used to control crossings, some vehicles have the right of way and the right of way over these systems. The superiority of the pass is that certain vehicle drivers are not bound by traffic restrictions and prohibitions while on duty, provided that they do not endanger the safety of life and property.

In general, an intelligent signaling system is developed in this study to solve this problem. Unlike similar studies, the developed system can be changed on the fly. The control of the system is in the hands of the person driving the emergency vehicle, and the driver can control the traffic lights from the scene to the destination point via the mobile application. From the light control page of the mobile application, a status message is sent to the light to be passed, indicating what its status should be. The traffic light module receives this message information, processes the message, and turns the light green when the vehicle passes. After passing through the activated traffic light, the return of the signaling to normal operation mode is again carried out by the person using the mobile application. In this way, there are no traffic jams in front of emergency vehicles and no loss of time while passing through traffic lights. The main contributions can be summarized as follows:

1. With these newly developed signaling systems, emergency vehicles will be able to reach their destination in a shorter time.
2. The safety of other vehicles in traffic will be ensured.
3. Idling vehicles emit more carbon dioxide into the air than moving vehicles. This system will reduce air pollution in the area where the system is operating. It will also avoid the noise pollution caused by vehicles honking their horns when the emergency vehicle arrives.
4. GSM infrastructure, which is widely used in smart home systems, can also be used in intelligent transportation systems and will contribute to applications in this area. Furthermore, the application of the methods and materials used in the developed signaling system will contribute to intelligent traffic systems and intelligent traffic light applications.

The remainder of this article is organized as follows: Section 2 reviews several recent studies of signaling systems developed for emergency vehicles. Section 3 explains the system, design, and development phases. Section 4 presents and discusses our experimental results for the proposed signaling system. Finally, Section 5 concludes and summarizes the paper’s findings and offers recommendations for future research directions.

## 2. Related Works

There are studies and conducted research in the literature for solving this and similar problems. When the research studies are examined, it is seen that the studies designed for the passage of ambulances and implemented on prototypes are generally encountered to solve this problem.

The authors of [11] developed a system that can operate while receiving signals from emergency vehicles due to radio frequency transmission. They used a Programmable Integrated Circuit (PIC) 16F877A microcontroller and a frequency of 434 MHz to return to normal operation after the emergency mode was activated.

The authors of [12] developed an Emergency Vehicle Signal Stop system (TJ-EVSP) based on vehicle-to-vehicle and vehicle-to-infrastructure systems called Collaborative Vehicle-Infrastructure Cooperation. They applied it to real traffic in Taicang City, China, and showed that it could improve the efficiency of emergency vehicle operations.

In [13], a study on the passage of an ambulance was carried out. The authors used radio frequency (RF) technology in their study. A new signaling system was provided by placing an RF transmitter in the ambulance and an RF receiver in the traffic system. After the signal from the ambulance was detected, information was sent to the PIC microcontroller controlling the traffic light. When the ambulance is detected, the light in the signaling system turns green. In the absence of an ambulance, the routine operation was ensured with signaling.

In [14], the authors calculate the distance between an emergency vehicle and an intersection using visual sensing methods. Manhattan, Euclidean, and Canberra distance techniques are used for distance calculation. They developed the PE-MAC protocol based on the MAC protocol to transmit the emergency vehicle’s information to the Traffic Management Center.

In study [15], which focuses on the problems faced by ambulances in traffic, signaling control is provided using RFID technology so that the ambulance can reach the target destination on a road with four intersections without traffic disruption. A receiver is placed at a certain distance from the signaling system. When the RFID placed in the ambulance was detected by the receiver, the traffic lights were controlled according to the timer. In addition, the system controls the lights according to the traffic density during routine working hours.

In study [16], the authors designed and developed an experimental setup that can access vehicle status and location information and mobile software that can share this information with other vehicles. The designed system was built in an experimental setup and tested on two vehicles. It used OBD-II (On-Board Diagnostics) compatible ELM327 and OPCOM diagnostic devices to access the in-vehicle communication network. The data rates for the connection between the mobile devices and the diagnostic device were measured, and the transfer rates of acquired data to the server were evaluated. In the event of a possible accident, drivers were warned by detecting surrounding vehicles within 1 km of the accident status information.

The authors of [17] placed ZigBee modules in emergency vehicles that communicate via radio frequency. When the ZigBee in the vehicle came within range of another ZigBee, they sent a message via radio frequency. The message was received by the ZigBee receiver and processed by the microcontroller to control the traffic light.

In this study [18], they proposed a traffic signalization system based on traffic density. The system automatically changes the signalization timing according to the traffic density at intersections. Their system is also configured with a camera. Images were captured with the camera, and the number of vehicles was calculated from the captured image. They used the masking algorithm for calculation and image processing. The camera also detects the siren of emergency vehicles and turns on the green light for these vehicles. They used the Arduino board for light control.

In [19], they discussed the problems faced by emergency vehicles in traffic in India and designed a traffic signaling system for emergency vehicles to overcome crises. In their system, they used Radio Frequency (RF) technology and an Arduino UNO board to clear the traffic and ensure that an emergency vehicle reaches its destination on time. In the designed signaling system, the transmitter module is placed in the emergency vehicle, and the receiver modules are placed in the traffic lights. When the button on the transmitter module in the emergency vehicle is pressed, the transmitter module sends a signal to the receiver module. After receiving the signal from the emergency vehicle, the Arduino activates the green signal to clear the road and allow the vehicle to pass.

In [20], they worked on a system that detects stolen vehicles and allows ambulances to move faster in busy traffic lanes. In the detection of stolen vehicles, the vehicle, whose information was entered into the database, was scanned through the module placed in the signaling system. As soon as the stolen car is detected, the traffic light turns red. In the system designed for the passage of the ambulance, when the ambulance arrives at a traffic intersection, it sends the emergency information through the RF transmitter, and the dynamic signaling system detects the information through the RF receiving sensor, and the light automatically turns green until the ambulance leaves the intersection and the lights in all other directions are red. The driver also sent the patient information to the hospital via SMS to the system.

The authors of [21] conducted a study for the project they developed, which aimed to alleviate their responsibilities towards emergency vehicles. First, they enabled these vehicles to communicate with other vehicles to create a route toward the destination; second, they communicated directly with signaling controllers to control the signaling system of emergency vehicles. This ensured that emergency vehicles did not run red lights and crossed intersections immediately on green lights. Finally, traffic information on the internet and information stored on the cloud storage platform were used to optimize the emergency vehicle’s route, and signaling controllers positioned along the emergency vehicle’s route were used to optimize the signaling program.

In [22], a system for controlling the traffic signal for emergency vehicles was developed through deep reinforcement learning, which provides fast emergency response in various scenarios and mitigates the negative impact of conflicting aspects on traffic efficiency.

In [23], they created a signaling system that will operate when it receives a signal from the emergency vehicle via radio frequency (RFID) transmission. They used a Programmable Integrated Circuit (PLC) Arduino microcontroller to normalize the operation of the lights before the emergency mode was activated. They used a frequency of 434 MHz to switch to an emergency light.

In [24], they developed a side lane application so an emergency vehicle can pass without waiting at traffic lights. Normal vehicles are allowed to pass through this side lane only when an emergency vehicle arrives. Priority assessment was performed for two emergency vehicles coming from different directions. Mathematical models were used in the evaluation.

Study [25] proposes a heterogeneous network model for connected Internet of Vehicles (IoV) and service-oriented network optimization, focusing on vehicle cloud, communication, and intelligent use cases as clients. A service-centric heterogeneous vehicular network model is proposed for connected traffic environments. Practical simulation and mathematical modeling of service-oriented network prioritization and content-centric service application in heterogeneous vehicular environments support the implementation of heterogeneous vehicular communication.

Priority vehicles cause major complications and accidents, especially when they use the right of way in signaling systems. In the proposed paper, we develop a new signaling system to solve this problem. The developed signaling system is controlled by the person in the emergency vehicle using a mobile application and SMS technology. First, the person driving the emergency vehicle creates a route between their current location and the destination location to check the traffic lights they will pass. On the route, there are traffic lights they will pass until they reach their destination. Before arriving at the traffic light to be crossed, the driver of the vehicle activates the light to be crossed from the control page. The driver first sends ON information to the module to which the traffic light to be passed is connected so that it turns green when the vehicle passes. At the same time, the OFF message is sent to the traffic lights in the other directions in the signaling so that these lights turn red when the vehicle passes. After the vehicle completes its passage, it normalizes the signaling operation after sending a message from the control page and the driver. The signaling system resumes its normal operation. This process is repeated for all signaling systems to be passed until the emergency vehicle reaches its destination. Our proposed system, together with other existing benchmarking systems. A comparison of similar works and our proposed system is given in Table 1.

## 3. System Description

This section provides information about the operation of the developed traffic lights.

### 3.1. System Overview

**Figure 1:**
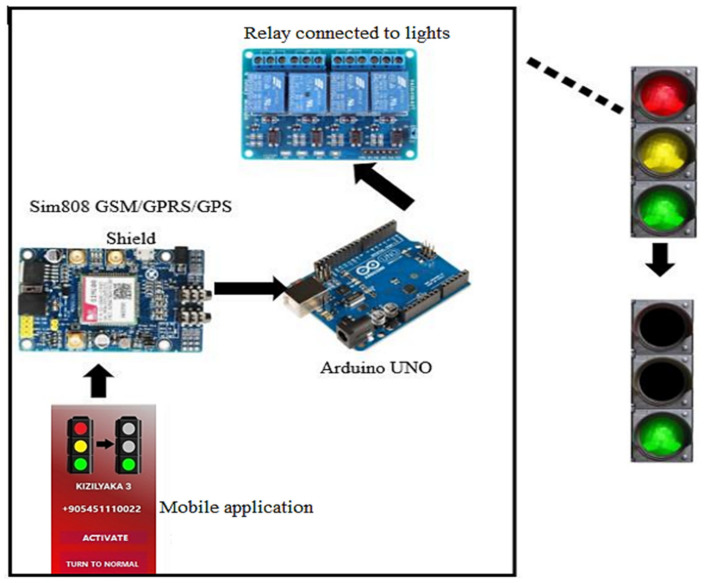
Overview of the proposed system.

An overview of the proposed system is given in Figure 1.

In order for the system shown in Figure 1 to work, the person using the emergency vehicle must first log in to the mobile application. The route is created after selecting the current location of the driver and the vehicle, and the target location. When the map is ready, signal lights are placed according to their location based on latitude and longitude information. A person has to select the first signal light to cross his route. In order for the selected light to turn green during the passage of the vehicle, click the “Activate” button on the control page. Here, an ON message is sent to the system to which the light on the vehicle’s route is connected. Sim808 GSM/GPRS/GPS module receives this message and transmits it to Arduino. The Arduino processes these data and controls the relay to which the lights are connected, and ensures that the light turns green during the passage of the vehicle. After the vehicle has passed, the operation of the traffic lights is normalized via the mobile application with a return to normal message sent by the driver from the mobile application.

### 3.2. System Flowchart

**Figure 2:**
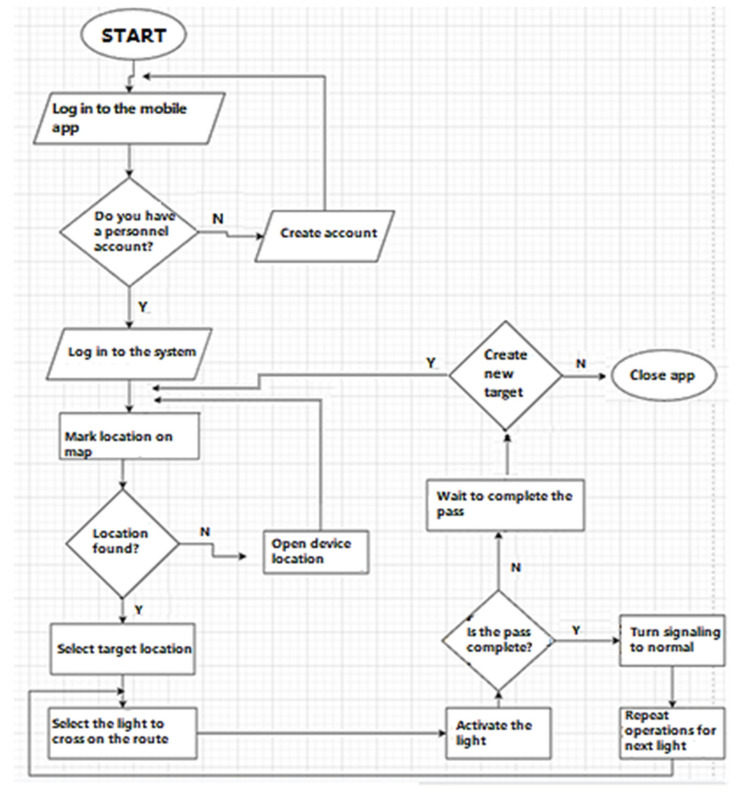
Flow chart of the system.

Figure 2 shows the flow chart of the system. After opening the system, the person driving the vehicle logs into the application. Upon entering the application, the location is marked on the map screen that opens. After selecting the destination, the route is created. Whatever traffic light is to be passed on the route, the driver selects that traffic light, and the light control screen opens. Here the traffic light is activated. After crossing, the signal returns to normal. The user repeats the same process for all traffic lights to be crossed until the destination is reached.

### 3.3. System Operation

**Figure 3:**
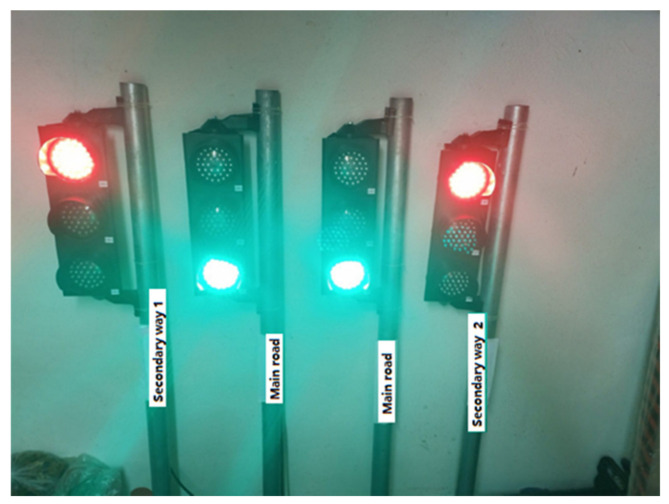
Traffic lights.

In this section, real traffic light circuits were created, whose operating conditions are shown in Figure 3.

**Figure 4:**
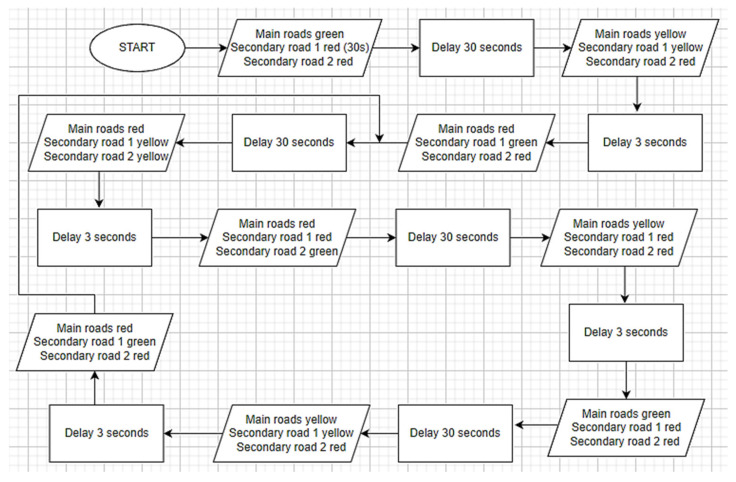
Flow diagram of the operation of traffic lights.

In the system, two traffic lights represent the main road; one light represents the first secondary road, and the other the second secondary road. On this type of road, the main roads cross each other while the secondary roads wait for the main roads to complete the crossing. After the main roads have completed their passage, there is a turn for the secondary roads. For example, when the first secondary road passes, the second secondary road has to wait. The sequence of traffic lights is as follows:

Traffic lights on main roads start with a green light. The duration of the green light is 30 s. Highways are kept green and secondary roads are kept red. In order not to interfere with the operation of the first secondary road and the operation of the second secondary road, one-time codes have been added to the codes of the first secondary road. The first operation of the first secondary road starts with these one-time codes. With these codes, it is ensured that the 1st secondary road lights are red for 30 s, yellow for 3 s, and green for 30 s. After the application of these lines of code, the first secondary road continued to operate for 70 s on red, 3 s on yellow, and 30 s on green. The operation of the system is given as a flow diagram in Figure 4. These durations were determined according to the flashing times of the traffic lights operating on the highway.

In order to control the system via the mobile application, it is necessary to wait approximately 70 s. The reason for waiting is that the codes we added for the first secondary path, which will run one time, have been completed. It is mandatory to complete the execution of these codes because when the command for light control comes from the mobile application, the codes running for the first secondary path cannot process the incoming command immediately because it uses the delay method. In order to process the command, all lines written with delay must be executed and finished. When the execution of code is not waited for, it causes complexity. After waiting for 70 s, there is no problem processing the commands from the mobile application since the entire system works with the millis() function.

After logging into the mobile application and creating a route, the traffic light to be passed through the light control interface is activated by clicking the “Activate” button, and the status information is sent as a message to the traffic light. The identity information on the interface is actually the information of the SIM card inserted in the SIM808 card to which the traffic light is connected.

**Figure 5:**
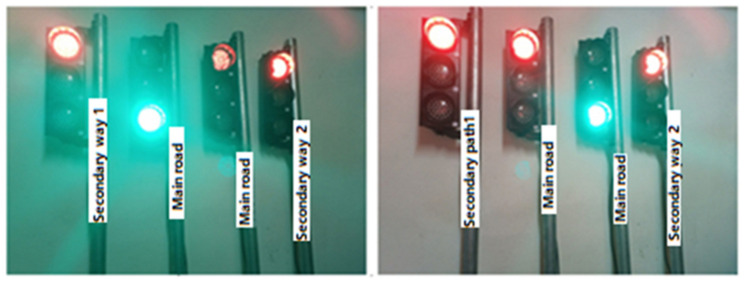
Active state of the lights on the main roads.

When the driver of the vehicle turns on the light, an “ON” message is automatically sent to this number so that the light turns green. In addition to this traffic light, the message “OFF” is automatically transmitted so that the lights in other directions turn red when the vehicle passes. No time is set for the lights to return to their normal operating mode. The normalization of the signaling operation after the crossing is completed is performed by the vehicle driver. The staff normalizes the operation of the signaling by clicking on the “Back to normal” button. At this stage, all traffic lights are automatically normalized by sending the “R” message. Figure 5 shows the activation of traffic lights on other roads for the passage of the vehicle coming from the main roads with the command sent from the mobile application.

**Figure 6:**
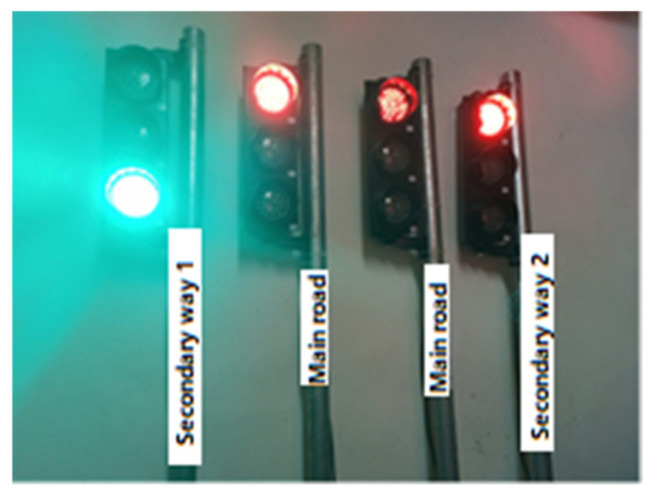
Activated state of lights on secondary way 1.

**Figure 7:**
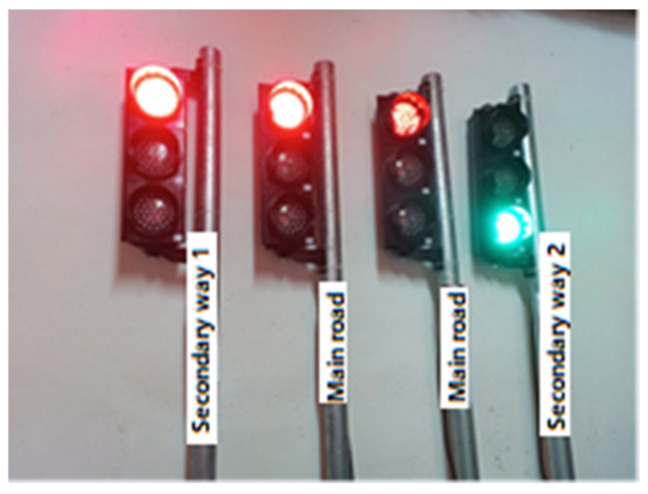
Activated state of lights on secondary way 2.

If the vehicle is coming from the first side road, the appearance of the lights will be as in Figure 6, and if the vehicle is coming from the second side road, the appearance of the lights will be as in Figure 7.

## 4. Installation of Traffic Lights and Mobile Application

This section provides information about the materials used in the developed system, the installation stages of the traffic lights, and the use of the mobile application.

### 4.1. Traffic Light Materials

Arduino UNO board, four-channel 5 V relay board, SIM808 GPS/GPRS/GSM module, triple traffic lights powered by 12/24 V, 12 V adapter, 9 V adapter, and 8 V adapter, a jumper for connection cable and female and male DC power supply cables are used in the installation of traffic lights in the signaling system. The properties and intended use of the materials used are given below, respectively.

#### 4.1.1. Arduino UNO

**Figure 8:**
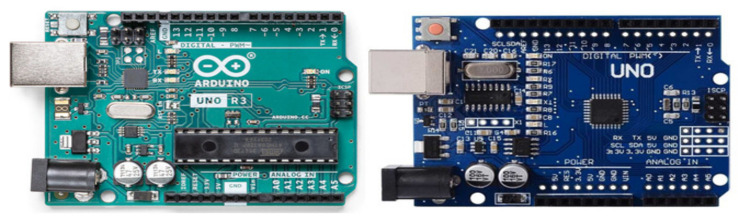
Arduino UNO original and Arduino UNO clone.

Among Arduino boards, the most useful and well-known Arduino Uno model [26]. All Arduino Uno models have 14 digital inputs (D0–D13), six analog inputs (A0–A5), a USB port, and an adapter port. The original models of the board have an Atmega328p microcontroller (Figure 8).

Arduino Uno can be operated by connecting to a computer via a USB cable, or it can be powered by an external power supply (battery, adapter) [27].

#### 4.1.2. SIM808 GPS/GPRS/GSM Development Module

The SIM808 card is a device that can be used for data communication between cellular network systems. This module supports quad-band GSM/GPRS communication operating at frequencies of 850 MHz, 900 MHz, 1800 MHz, and 1900 MHz [28,29]. It provides ease of use in projects thanks to its small size and lightweight. There are SMA connectors on the board for antenna connections. Separate antennas must be used for GSM, GPS, and Bluetooth. To initialize the board, press and hold the “start” button for 2 s after connecting the power. The network connection status of the card can be monitored from the LEDs on the board. It has a 1.25 V, 3.3 V, and 5 V compatible TTL serial connection (UART) for connecting development boards such as Arduino and Raspberry Pi [30].

#### 4.1.3. Four-Channel 5 V Relay Board

**Figure 9:**
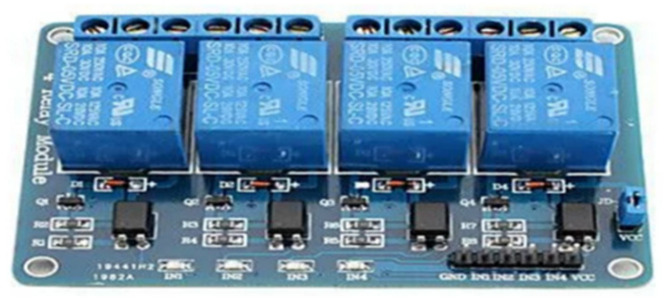
Four-channel 5 V relay board.

A relay is a programmable electrical switch controlled by an Arduino board or any microcontroller (Figure 9). It is used to programmatically control or operate devices with high voltage and/or current. It is a bridge between Arduino and high-voltage devices [31]. There are three connections on the relay, usually labeled NO (normally open), NC (normally closed), and COM (common). The normally open connection is a common-ended open circuit when the relay is not energized, so no conduction occurs. When the coil of the relay is energized, the NO and COM terminals are short-circuited, allowing electric current to flow. The NC terminal works in the opposite direction to the NO terminal, i.e., when the coil is not energized, the NC and COM terminals are shorted. When energized, they form an open circuit. COM (Common) is the common input. To this input [32], the voltage source that the relay will switch is connected.

#### 4.1.4. 12/24 V Traffic Light

**Figure 10:**
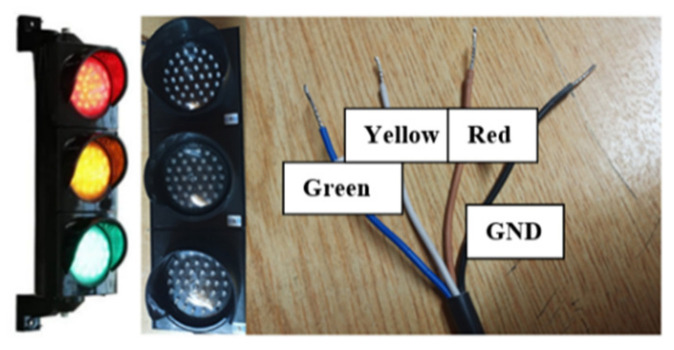
Traffic light and pinouts.

Developed in 1912, traffic lights are signaling devices designed to control traffic flow at road intersections, pedestrian crossings, railway trains, and other places. There are four different outputs in traffic lights used in signaling construction. These are the control outputs of red, yellow, and green LEDs and the ground (GND) output (Figure 10).

### 4.2. Prototype

Before the physical installation of the signaling system, a prototype of the system is created. This prototype reflects the similar operation of the real system.

**Figure 11:**
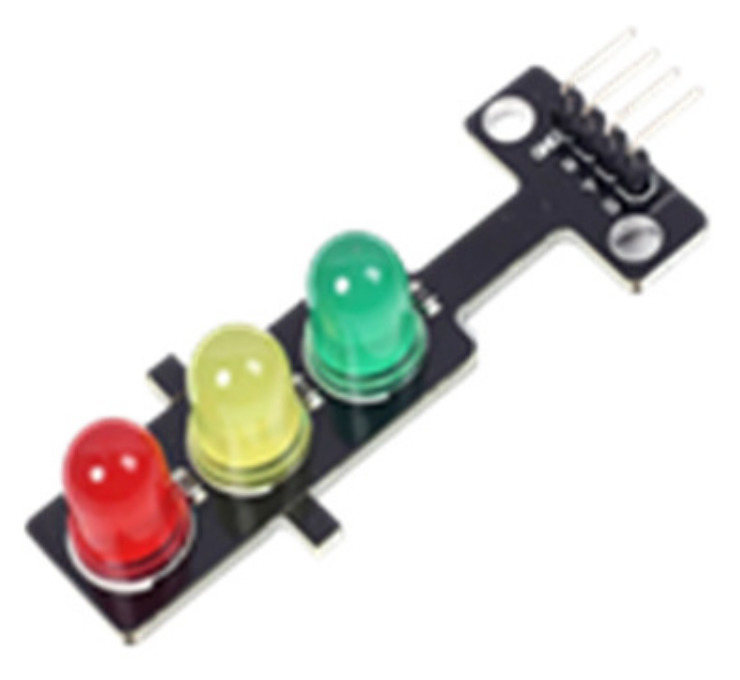
Traffic light modüle.

Errors in the software were eliminated in the trials made on the prototype. Arduino Uno, Sim808 GSM/GPRS/GPS card, and traffic light module were used to prototype the system. Since the supply voltage of the traffic light modules is 5 volts, no relay was used in the prototype. The traffic light module has three pins (red, yellow, and green) and one ground (GND) pin. A visual representation of the module is given in Figure 11.

**Figure 12:**
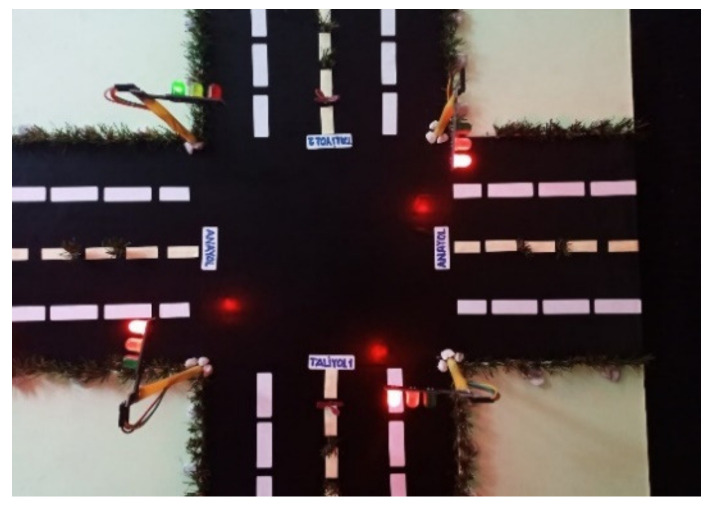
Top view of the prototype.

**Figure 13:**
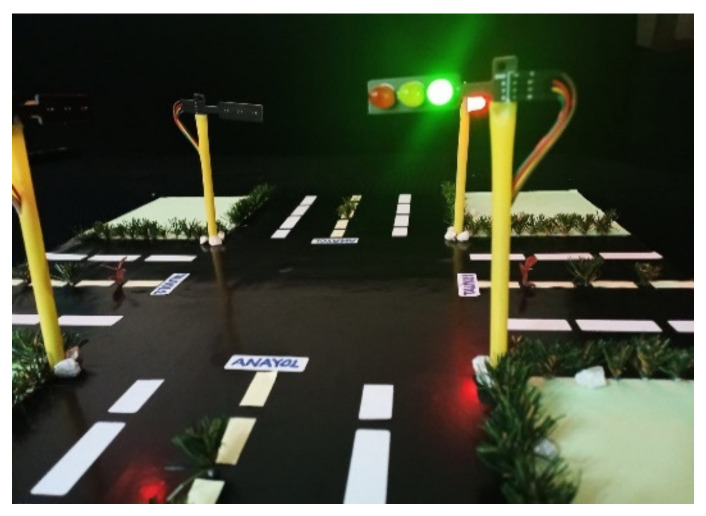
Side view of the prototype.

The top view of the prototype is shown in Figure 12, and the side view is shown in Figure 13.

### 4.3. Hardware Design

Circuit designs were made using Fritzing software in the setup of the system. Fritzing is open-source software that enables the original development of electronic circuits using Arduino boards. Fritzing users can prepare prototypes of the circuits and circuit elements they develop, share them with others, and use their outputs. It is possible to use experimental circuit boards, schematics, printed circuits, and code related to Arduino in the Fritzing program. The circuit elements are divided into sections and are categorically accessible [33].

#### 4.3.1. Connection of Arduino UNO and SIM808 GSM/GPRS/GPS Board

**Figure 14:**
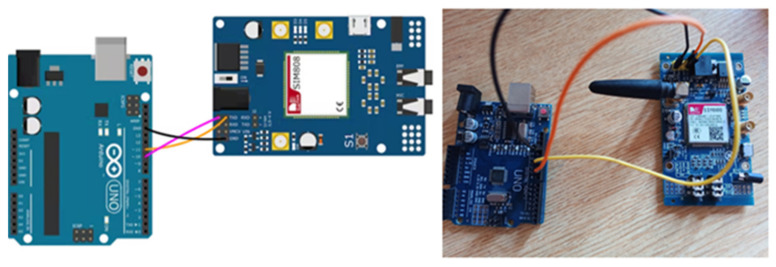
Connection circuit design and actual connection.

When connecting the Sim808 GSM/GPRS/GPS board to Arduino UNO, the UART TTL interface on the Sim808 board is used. Three pins are used for connection in this section. These pins are RX, TX, and GND pins. A connection diagram and the actual connection are given in Figure 14.

#### 4.3.2. Connection of Relay and Traffic Lights

**Figure 15:**
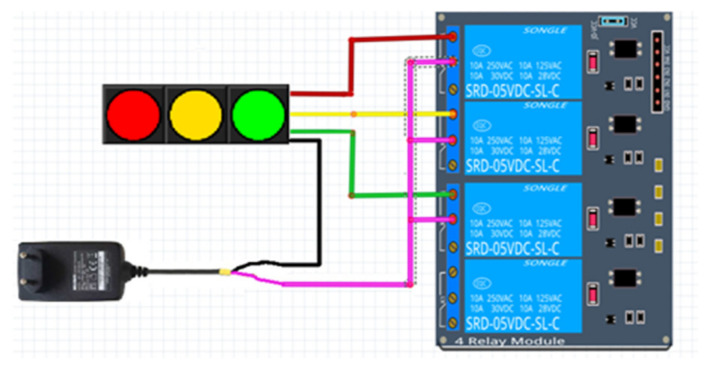
Traffic light and relay connection.

There are four cables in the traffic lights used in the installation of the system. Three belong to colors, and one to ground (GND). The lights work between 12 and 24 volts. Since there are three LEDs to be controlled in traffic lights, four-channel relays are used. When connecting the lights with the relay, the cables to which the LEDs are connected are connected to the NO pin of the channels on the relay. The COM pins of the relay to which the LEDs are connected are also connected to each other and connected to the power supply with the help of a terminal. The GND cable from the light is connected to the power supply used. The connection diagram is given in Figure 15.

If the proposed system is to be installed at real traffic lights for a long time, it needs to be fed with a constant power supply. This will be provided by a solar panel mounted on the pole.

#### 4.3.3. Arduino Uno and Relay Connection

**Figure 16:**
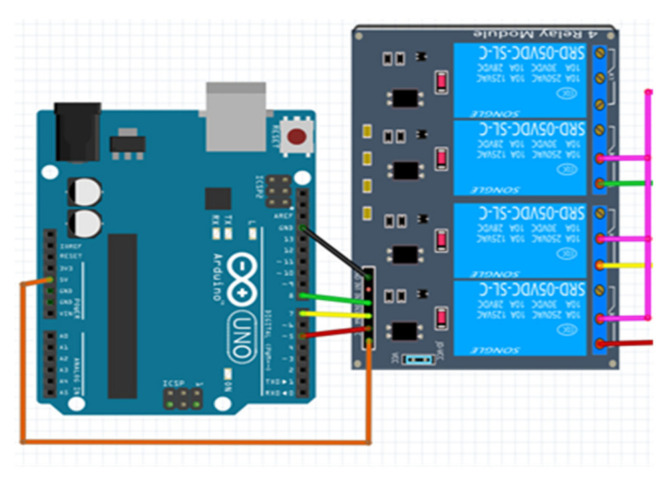
Arduino UNO and relay connection.

By using a relay module with an Arduino board, it is possible to turn on and off devices operating with AC or DC voltage [34]. Relay modules are sold with 5 V or 12 V power supply. In this project, since a maximum of 5 volts of power can be obtained from an Arduino UNO, a relay with an operating voltage of 5 volts was used. When connecting the relay to the Arduino UNO, the VCC, GND, and signal pins on the front of the relay module are used. The 5 V relay module is powered by the 5 V pin on the Arduino UNO. At the signal end, the lights are controlled by sending a signal from Arduino UNO according to the incoming message information. The connection of the relay with Arduino UNO is given in Figure 16.

The most important point to be considered when connecting Arduino and relay is the connection of signal pins. The ones corresponding to the channels connected with the signal pins should be used. The connections of K2, K3, and K4 channels are made in the connection diagram in Figure 16. The K2 channel corresponds to the IN2 pin, the K3 channel corresponds to the IN3 pin, and the K4 channel corresponds to the IN4 pin.

### 4.4. Mobile Application

The developed mobile application is designed for the person using the emergency vehicle. The use of the application belongs only to the relevant person. The mobile application has a login screen, user registration screen, map screen, and traffic light control screen. The necessary information to access the application is given to the person using the vehicle by the organization they are affiliated with. This login information is saved in the Firebase real-time database.

**Figure 17:**
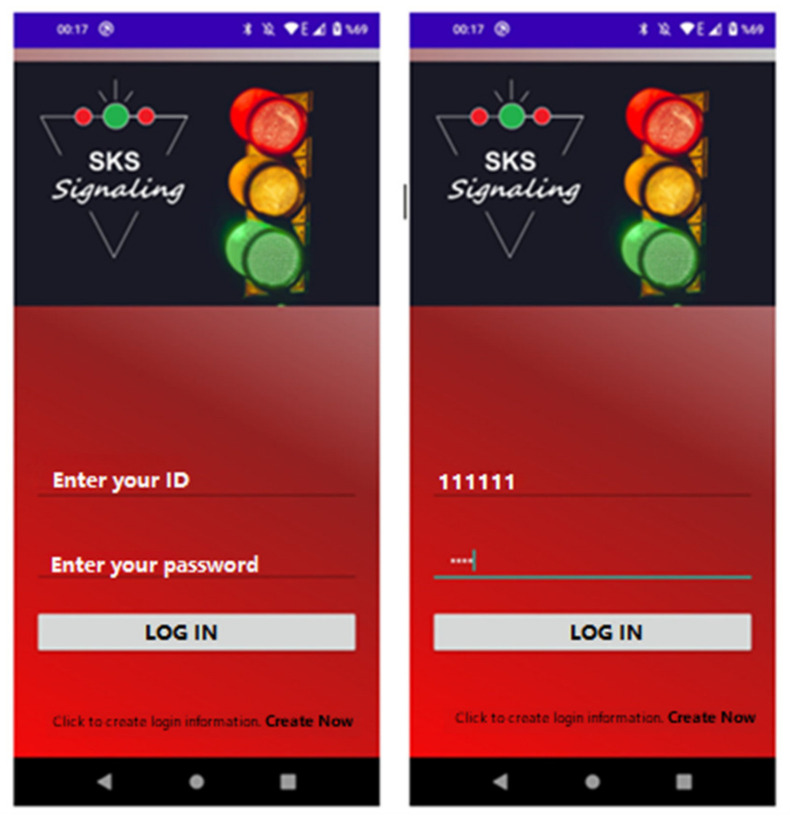
Login page and information entered.

It is possible to create a new account by logging in from the login screen and switching to the next screen or another screen. On this screen, the person logs into the system using the ID and password information given to them or the information they create themselves. The login information of authorized personnel is previously saved in the Firebase database. The user’s login information is verified with Firebase Authentication. After the login information is verified, the message “Login Successful” appears on the screen. The login screen is given in Figure 17.

**Figure 18:**
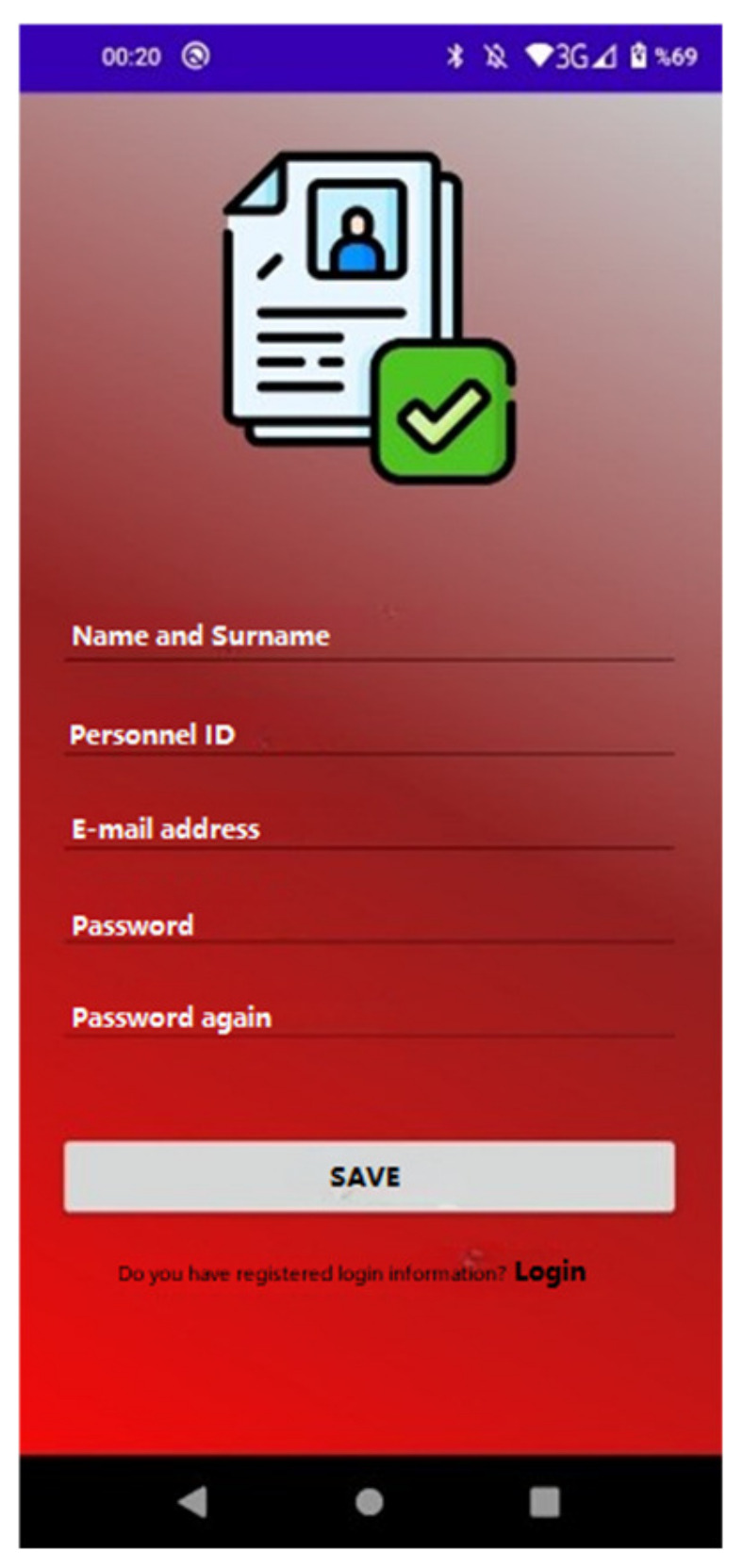
Person registration screen.

If the login information is not given to the person, they can go to the registration screen in Figure 18 by clicking the “Create Now” button at the bottom of the login page. After the user enters the required information on this screen, the user is automatically directed to the login page after the “Contact Registration Successful” notification message appears on the screen.

**Figure 19:**
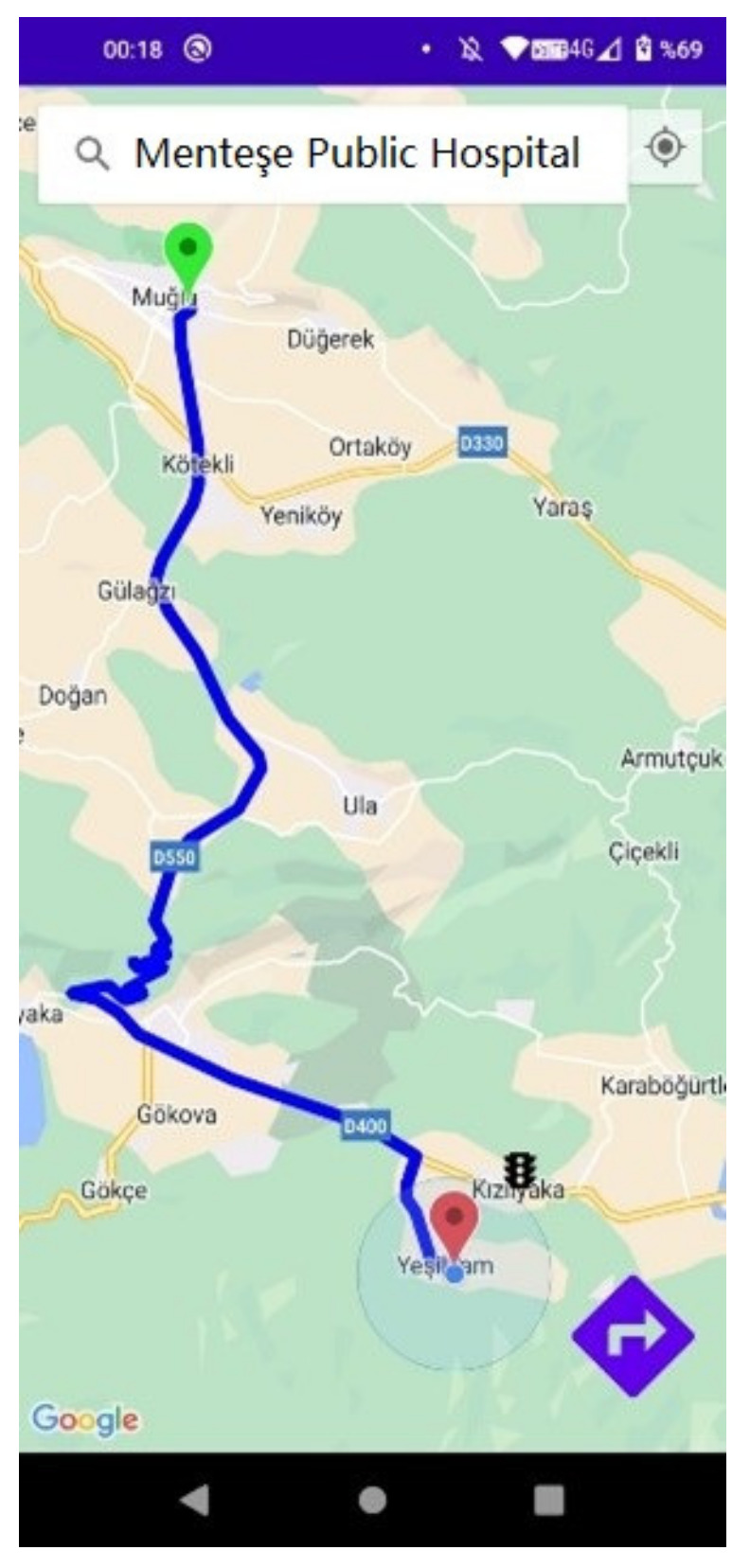
Route creation screen.

After logging in to the account, the screen in Figure 19 welcomes the person. On this screen, the person can create a route between the destination location and the current location. As soon as you log in to the system, the current location is marked on the map with a red marker. If the location is not marked automatically, the current location can be marked on the map with the button on the top right corner of the interface. On the other hand, the destination location can be selected from the drop-down list by entering the name of the location in the search tab at the top of the interface.

**Figure 20:**
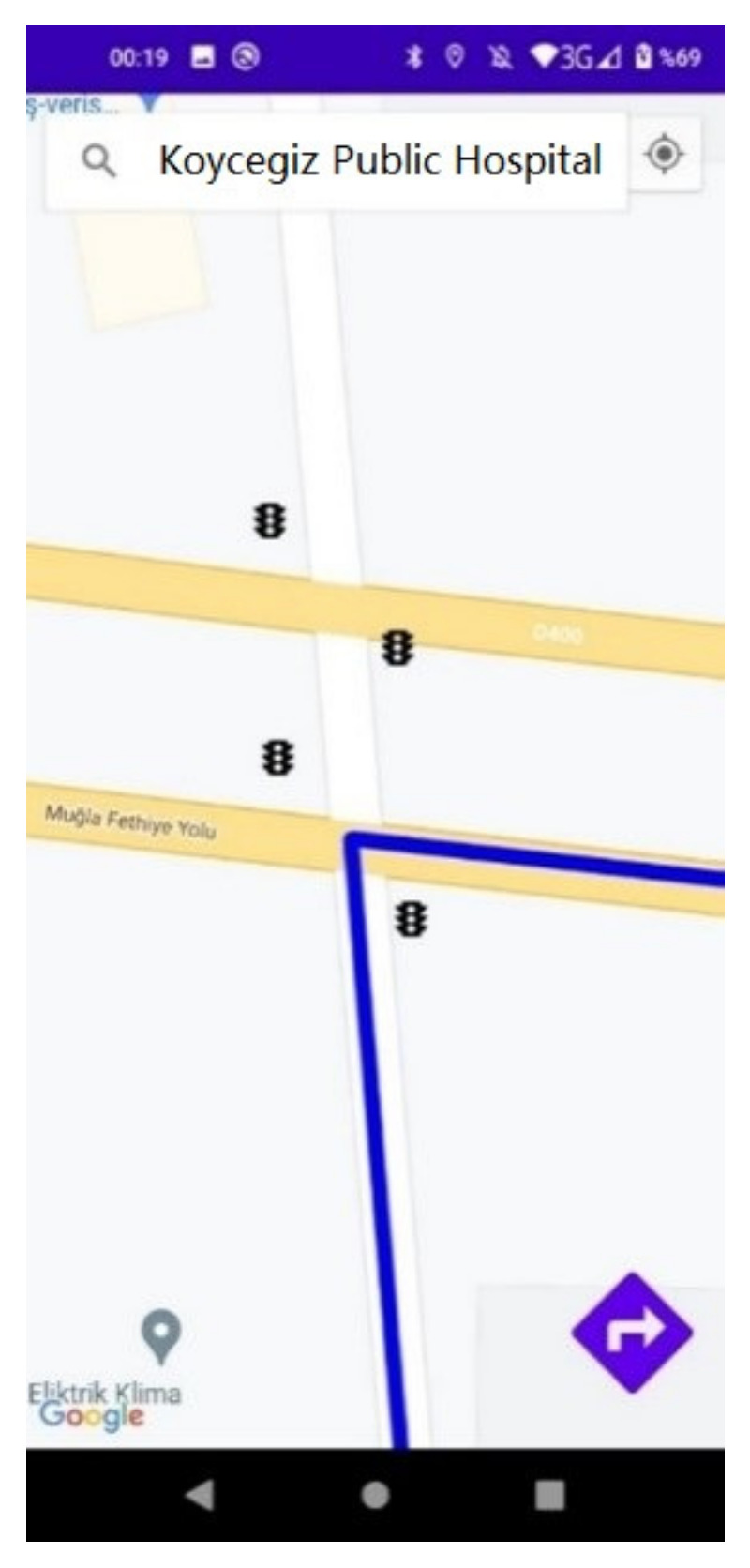
New route creation and traffic lights on the map.

The Google Directions API is used in Android applications to create routes between two specified points. To create a route, the API key information and the latitude and longitude information of both the current location and the destination location are sent via a URL to the Google Maps Directions web service. When a new destination is entered, the previously plotted route is deleted, and a new route is created. In Figure 20, a new location is entered, and a new route is created. A close-up view of the traffic lights placed on the roads is also given by zooming in on the map.

**Figure 21:**
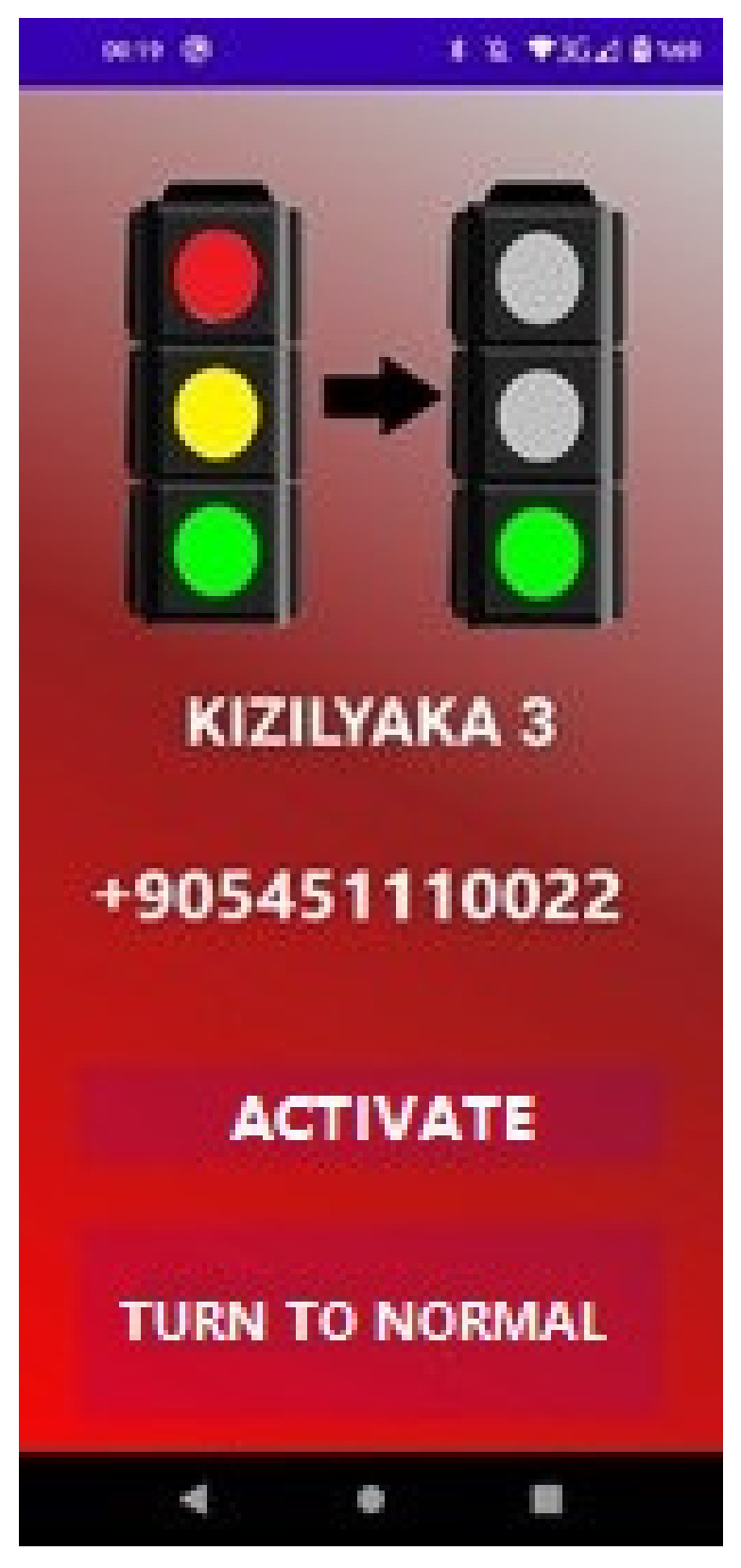
Traffic light control page.

After creating the route on the previous page, the traffic light in the direction of the vehicle must be selected in order to pass the first signalization encountered. When this traffic light is selected, the page where the light will be controlled opens (Figure 21).

**Figure 22:**
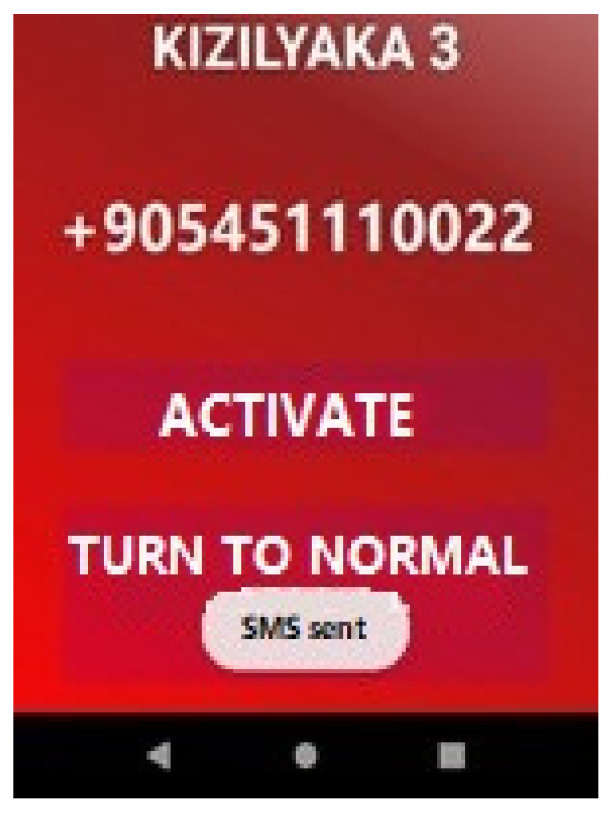
Printing the “SMS sent” message on the screen.

This screen contains the name of the light to be passed, the number of the module to which it is connected, and buttons to control the light. With the “Activate” button on the interface, SMS information is sent to all lights in the signaling area about what their status should be during the passage of the vehicle. The “ON” message is sent to the traffic light module that the vehicle will pass, and the “OFF” message is sent to the traffic lights in other directions. The numbers to send the message are retrieved from the database. If SMS sending is successful, the “SMS sent” information message is printed on the screen (Figure 22). When SMS sending is unsuccessful, the information message “SMS could not be sent” is printed on the screen. If there is an error with permissions, “General Error” appears on the screen; if there is no service on the phone, “No Service”; if there is a failure because a PDU (Protocol Data Unit) is not provided, “No PDU;” and if the phone has airplane mode, “Airplane Mode On” is printed on the screen.

In order to send messages, SMS sending permission must be added to the AndroidManifest.xml file. In addition, in order for the application to send SMS automatically, SMS sending must be allowed in the mobile application.

## 5. Results and Discussion

In this section, the operation of the developed traffic lights was tested. In addition, the number of emergency vehicles passing through the signaling system, the total number of vehicles passing through the signaling system, and the distance that other vehicles should travel during the arrival of the emergency vehicle in case of failure of the developed system were determined.

### Field Test

**Figure 23:**
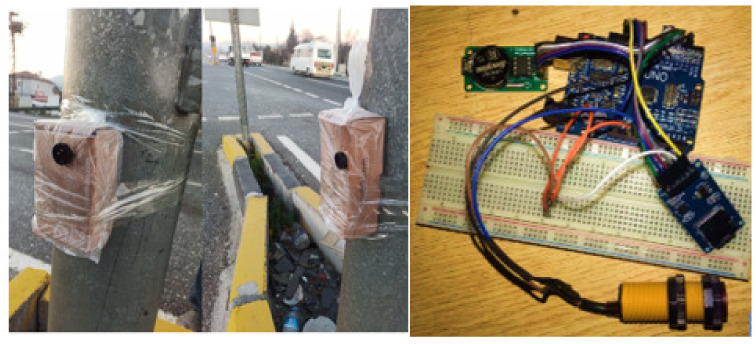
Vehicle counter modules.

With the module installed using Arduino Uno, SD card module, DS1302 RTC module [35], and MZ80 infrared sensor [36], the number of vehicles passing through the routes determined in the field study was counted. The installed devices and their installed state are given in Figure 23. In the module installed for vehicle counting, the MZ80 infrared sensor is used to detect obstacles and objects in front of it. DS1302 RTC module is used to attain time information. Data obtained are written to the SD card inserted in the SD card module.

**Figure 24:**
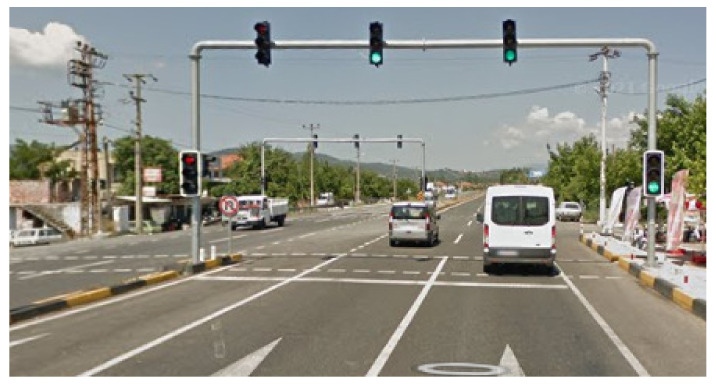
Mugla-Fethiye route.

**Figure 25:**
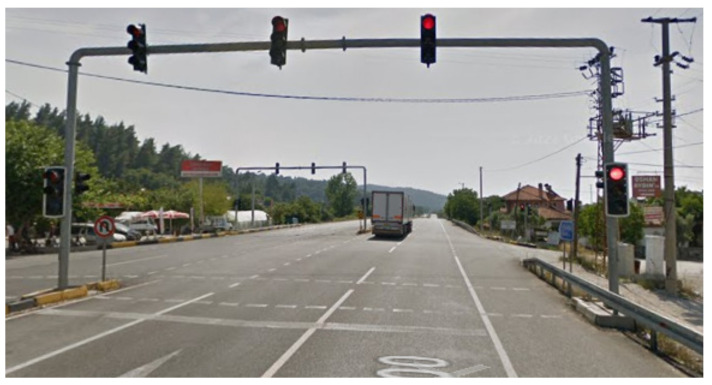
Fethiye-Mugla route.

In this section, the vehicles used in the existing signaling system are examined in order to study the operation of the signalization system installed in the field test in Muğla province of Turkey. Investigations were carried out on the Muğla-Fethiye and Fethiye-Muğla routes of Muğla Province, including the signalized intersection shown in Figure 24 and Figure 25.

The field tests were carried out on Thursday between 08:30 and 10:30 on the busiest day of the week. Data obtained during these hours are given in Table 2.

When Table 3 is analyzed, the passage of normal vehicles and emergency vehicles mostly took place on the Fethiye-Muğla route. This is because this route is used for transportation to the city center, headquarters, and the university hospital. In addition, another reason for the crowding is the bazaar in the city center on Thursdays. The number of vehicles waiting at red lights during the passage of emergency vehicles on the Fethiye-Muğla route is given in Table 3.

**Figure 26:**
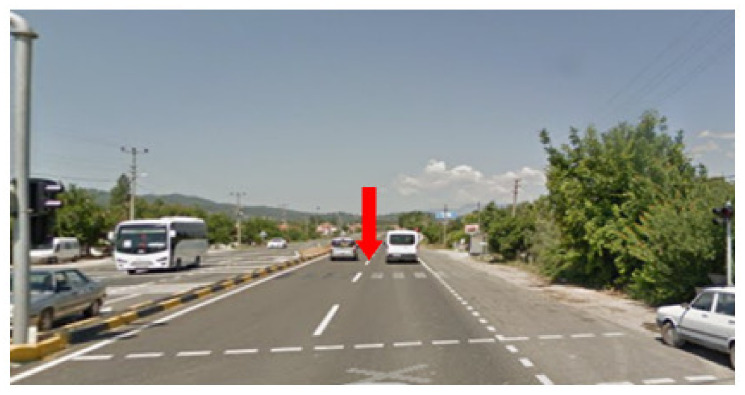
The point where vehicles accelerate.

On the prototype, a SIM card was inserted into the module to which the traffic light was connected in the same direction, and this traffic light was activated from the mobile application. An SMS with the status of the traffic light was sent to the module to which the traffic light was connected in about 5 s. This 5 s delay refers to the time it takes until the SMS information sent from the mobile application is received from the SIM808 module and processed on the Arduino Uno. No distance is specified for the control of traffic lights. However, it was determined that in order for the emergency vehicle to easily pass through the traffic light, a minimum distance was needed for other vehicles waiting at the light to accelerate and clear the way. It was determined that the vehicles waiting at the light would need to travel approximately 92 m to accelerate and clear the road. When determining this value, the average of the points at which moving vehicles accelerate after the traffic light turns green is taken into account. The point at which the vehicles accelerate is shown in Figure 26 with a red arrow.

When vehicles reach this point, it creates a suitable passing area for the emergency vehicle to pass. There is no specific distance between the emergency vehicle and the traffic light. This is because the number of vehicles waiting at traffic lights varies. The driver of the vehicle should adjust when the traffic light is activated, taking into account this distance value and the density of traffic.

## 6. Conclusions

In this study, the GSM infrastructure used in smart home systems is preferred for remote control of traffic lights. An Arduino Uno board was used for control. The personnel driving the emergency vehicle send the status information of the traffic lights to the Arduino Uno board, to which the traffic lights and the SIM808 module are connected via the mobile application. After the passage of the emergency vehicle was completed, the operation of the signaling system was returned to normal via the mobile application.

Compared to studies in the literature, systems containing circuit elements such as sensors or cameras are more likely to be damaged in adverse weather conditions or on-road routes.

The difference between our proposed system from other systems described in the literature is that internet infrastructure is not used to control traffic lights. If internet infrastructure were used to control the lights, this system would not be effective in some areas where there is no internet infrastructure. Another reason for not using these technologies is that there are many factors that affect and weaken the signals or radio waves. Various buildings in urban areas, trees in rural areas, electronic devices, antennas, etc., are among these factors and can be seen on the move in the environment and atmosphere through which the signals and radio waves pass. The internet connection is used to log on to the proposed system and establish a route between the current location and the destination location. This is mobile internet. No external internet infrastructure is needed for traffic signaling. Disconnecting the internet connection after user authentication and routing is complete does not prevent traffic lights from being controlled because the number information of the modules to which the traffic lights are connected is retrieved from the database and assigned to the relevant variables on the light control page. In this way, traffic lights can be controlled offline from the light control page.

If the system encounters a problem while sending an SMS message, a notification message is immediately displayed to the user, explaining the problem. Error logs are analyzed on the development platform, and possible errors that may occur in the operation of the application are eliminated.

In the system set up as part of the study, the traffic lights were to be controlled depending on the distance between the vehicle’s location and the location of the traffic lights, but this could not be realized because the Google Cloud console components used do not support real-time location. Instead, the GSM component of the SIM808 card was used to control the traffic lights via SMS.

In future studies, the position of the vehicle will be monitored immediately via the mobile application, and the traffic light will be activated if the distance between the traffic light and the traffic light to be crossed falls below a certain value. If several emergency vehicles approach the intersection at the same time from overlapping directions, the system provides sequential passage according to the distance of the vehicles from the traffic light, the degree of urgency of the emergency vehicle, and the type of emergency vehicle. If the user forgets to activate the traffic lights, the traffic lights can be activated with the help of a camera mounted at a certain distance. This system is recommended for use by the competent authorities responsible for the management of emergency vehicles in the city.

## Figures and Tables

**Table 1:** Comparison of the proposed system with similar studies.

Ref	Year	Materials	Experiment Environment and Platforms Used	Results
[11]	2013	PIC 16F877A microcontroller	Prototype	Delay of emergency vehicles is prevented.
[12]	2013	DSRC (Dedicated Short Range Communication) devices, touch screen, OBD (On Board Diagnostic) interface, GPS receiver, RSU(Road Side Unit)	Linux, Microsoft Visual Studio, GMap.NET, Google, Yahoo!, Bing, ArcGIS	Field test results showed that the TJ-EVSP has the ability to increase the efficiency of emergency vehicle operations.
[14]	2016	Cameras	Matlab NS-2 Simulator	It has been determined that the protocol that sends the vehicle information the fastest is the PE-MAC protocol they recommend.
[17]	2018	ZigBee, RFID, microcontroller	No platform information used	The delay of the emergency vehicle is inhibited.
[24]	2022	RSU, 5G devices, sensors that exchange data, control units used in road management	No platform information used	The use of the side lane was effective, and the mathematical modeling steps used for the priority of emergency vehicles coming from different directions gave successful results.
Proposed System	2023	Arduino Uno, SIM808 GSM/GPS/GPRS, relay, 12/24 V traffic light	Prototype Newly installed traffic lights Arduino Android Studio	The traffic light that the vehicle would pass through was activated, and the other traffic lights turned red. The operation of the system was restored to normal after the migration was complete.

**Table 2:** Field test data.

Route	Total Number of Passing Vehicles	Total Number of Emergency Vehicles Passed
Muğla-Fethiye	550	Ambulance:1
Fethiye-Muğla	611	Ambulance:3 Police:2 Firefighter:1

**Table 3:** Number of Vehicles Waiting at the Red Light.

Emergency Vehicles	Number of Vehicles Waiting at the Red Light
Ambulance 1	8
Ambulance 2	4
Ambulance 3	5
Police 1	4
Police 2	5
Firefighter 1	6

## Data Availability

Not applicable.
